# 3,4-Methylenedioxy-β-Nitrostyrene Alleviates Dextran Sulfate Sodium–Induced Mouse Colitis by Inhibiting the NLRP3 Inflammasome

**DOI:** 10.3389/fphar.2022.866228

**Published:** 2022-06-15

**Authors:** Juanjuan Zheng, Zhongxin Jiang, Yue Song, Shu Huang, Yuzhang Du, Xiaobao Yang, Yan Xiao, Zhihui Ma, Dakang Xu, Jing Li

**Affiliations:** ^1^ Department of Clinical Laboratory, The Affiliated Hospital of Qingdao University, Qingdao, China; ^2^ Department of Inspection, The Medical Faculty of Qingdao University, Qingdao, China; ^3^ Department of Laboratory Medicine, Ruijin Hospital, Shanghai Jiaotong University School of Medicine, Shanghai, China; ^4^ Department of Medical Technology, Qiqihar Medical University, Qiqihar, China

**Keywords:** NLRP3 inflammasome, 3,4-methylenedioxy-β-nitrostyrene, IL-1β, experimental colitis, inflammatory bowel disease

## Abstract

Inflammatory bowel disease (IBD) has been reported to be associated with NLRP3 inflammasome activation. Therefore inhibiting inflammasome activation could be a new approach to treat IBD. Inflammasome inhibitors NLRP3-IN-2, JC124, and 3,4-methylenedioxy-β-nitrostyrene (MNS) were previously reported to exert anti-inflammatory effects in various disease models but not in the dextran sulfate sodium (DSS)–induced colitis model. Here, we showed that MNS was more efficient in inhibiting the secretion of interleukin-1β (IL-1β) by blocking oligomerization of apoptosis-associated speck-like protein (ASC) than NLRP3-IN-2 and JC124. To investigate the protective effects of MNS on enteritis, we administered intragastric MNS to DSS-induced colitis mice. The results demonstrated that MNS attenuated DSS-induced body weight loss, colon length shortening, and pathological damage. In addition, MNS inhibited the infiltration of macrophages and inflammatory cells and reduced IL-1β and IL-12p40 pro-inflammatory cytokines but had no significant effect on tumor necrosis factor α (TNF-α) and IL-6. Furthermore, we also found that the differentiation of IL-17A^+^interferon-γ (IFN-γ)^+^CD4^+^ T cell was decreased in the colon after MNS treatment, which might be mediated by IL-1β, etc. cytokine release. Taken together, MNS alleviated DSS-induced intestinal inflammation by inhibiting NLRP3 inflammasome activation, which may function as an effective therapeutic for IBD.

## Introduction

Inflammatory bowel disease (IBD), including Crohn’s disease and ulcerative colitis, is a chronic and intractable inflammatory disease of the gastrointestinal tract (Asquith and Powrie, 2010). The condition is believed to be triggered by environmental factors in genetically susceptible individuals. Beyond this, it is related to an impaired mucosal barrier system and dysregulated intestinal immune responses (Famularo et al., 2002; Kanneganti et al., 2007). The clinical symptoms of IBD include recurrent abdominal pain, diarrhea, and mucopurulent bloody stool, and it severely affects the quality of an individual’s life and increases the risk of colon cancer (Kim and Chang, 2014). Some IBD symptoms can be controlled by conventional drugs such as sulfasalazine and infliximab. However, these drugs are usually accompanied by a relapse of IBD (Patel et al., 2016; Wu et al., 2020). The reagent for anti–tumor necrosis factor α (TNF-α) may elicit good clinical effects, but it is not widely used because of the high cost (Cote-Daigneault et al., 2015; Subramanian et al., 2017). Therefore, it is urgent to find a safe and effective approach for IBD.

To understand dysregulated intestinal inflammation in IBD, an increasing number of recent studies have focused on inflammasome-mediated colitis. In 2002, Martinon et al. identified inflammasomes for the first time and stated that they comprise caspase, ASC, and NACHT leucine-rich-repeat protein 1 (NALP1) (Martinon et al., 2002). Since then, NLR family, pyrin domain containing 3 (NLRP3); NLR family, pyrin domain containing 1 (NLRP1); absent in melanoma 2 (AIM2); NLR family CARD domain-containing protein 4 (NLRC4); interferon alpha-inducible protein 16 (IFI16), and other inflammasomes have been identified one after another (Rock et al., 2010). Among the NLR family, the NLRP3 inflammasome has been studied more extensively due to its key role in the immune system and inflammatory diseases (Agostini et al., 2004; Zhen and Zhang, 2019). NLRP3 inflammasome activation could induce the secretion of pro-inflammatory cytokines, including interleukin-1β (IL-1β) and interleukin-18 (IL-18) (Zhivaki and Kagan, 2021). In patients with IBD, the NLRP3 inflammasome was activated, followed by increased mRNA and protein levels of IL-1β, caspase-1, and NLRP3, which in turn aggravated the severity of IBD (Mahida et al., 1989; Lazaridis et al., 2017). Bauer et al. found that the protein levels of IL-18 and IL-1β from macrophages of DSS-induced wild-type mice colitis were significantly higher than those of mice lacking NLRP3, ASC, and caspase-1 (Bauer et al., 2010). All of this suggests that the NLRP3 inflammasome plays a critical role in intestinal inflammation.

Although the NLRP3 inflammasome acts on a variety of human diseases, its pathogenesis is not fully understood. Therefore, specific NLRP3 inflammasome inhibitors can be used as pharmacological tools to investigate its pathogenic mechanisms and as potential therapeutic approaches. Among these small molecules that inhibit the inflammasome signaling pathway, MNS has previously been reported to promote wound healing by inhibiting the activation of the NLRP3 inflammasome and inhibiting the platelet Glycoprotein IIb/IIIa activation with the classical enteritis drug sulfasalazine (Patel et al., 2012). NLRP3-IN-2 and JC124, chemically synthesized sulfonamide analogs, could inhibit the formation of the NLRP3 inflammasome in cardiomyocytes and limit the infarct size following myocardial ischemia/reperfusion in the mouse, without affecting glucose metabolism (Marchetti et al., 2014; Fulp et al., 2018). To date, there are no results showing the effect of these three inhibitors in colitis.

In this study, we first compared the inhibitory effects of three inhibitors on IL-1β release *in vitro*. Then, we chose MNS, which was more efficient in inhibiting the secretion of interleukin-1β (IL-1β) and explored its efficacy in an experimental enteritis mouse model as it showed better anti-inflammatory effects and IL-17A^+^ interferon-γ (IFN-γ)^+^CD4^+^T cell–mediated intestinal immune responses. Thus, we proposed MNS as a new therapeutic agent that could promote IBD remission and maintain intestinal immune balance.

## Materials and Methods

### Reagents

NLRP3-IN-2 (4-(2-(5-chloro-2-methoxybenzamido)ethyl)benzenesulfamide), JC124 (5-chloro-2-methoxy-N-(2-(4-(N-methylsulfamoyl)phenyl)-ethyl)benzamide), and MNS (3,4-methylenedioxy-β-nitrostyrene) (purity >99%) were purchased from MedChemExpress (New Jersey, United States).

IL-1β, IL-6, IL-12, and tumor necrosis factor-α (TNF-α) enzyme-linked immunosorbent assay (ELISA) kits were purchased from Thermo Fisher Scientific (Waltham, United States).

Antibodies against NLRP3 (ab270449), ASC (ab175449), IL-1β (ab254360), MUC-2 (ab272692), and HRP Anti-Rabbit IgG (ab288156) were purchased from Abcam Systems (Cambridge, United Kingdom). Antibodies against claudin1 (13050-1-AP) were purchased from Proteintech (Chicago, United States). Antibodies against caspase-1 (24232S), P65 (8242S), P-P65 (3033), β-actin (4970), GAPDH (5174), and Ly-6G (87048) were purchased from Cell Signaling Technology (Boston, United States).

Nigericin (tlrl-nig), ATP (tlrl-atp), MSU (tlrl-msu), and silica (tlrl-sio) were purchased from InvivoGen (San Diego, United States). Disuccinimidyl suberate (DSS) was purchased from MP (Waltham, United States).

Flow cytometry antibodies, FITC-CD45 (109806), Pe-Cy5-CD4 (100540), APC-F4/80 (123116), PE-IL-17A (506904), APC-IFN-γ (505809), PerCP-Cy5.5-CD11b (101228), and the Zombie dye kit (423106), were purchased from Biolegend (San Diego, United States). Fixation/permeabilization buffer and permeabilization buffer were purchased from BD Bioscience (Franklin Lake, United States).

### Cell Culture

Bone marrow cells were collected from C57BL/6 mice by flushing dissected tibias and femurs with aseptic PBS. The cells were cultured for 7 days in DMEM with 10% fetal bovine serum and 1% penicillin–streptomycin. 30% L929 cell-conditioned medium was added to promote bone marrow–derived macrophage (BMDM) differentiation. The cells were cultured in a humidified incubator with 5% CO_2_ at 37°C.

### Cell Viability

BMDMs were plated in 96-well plates (5.0 × 10^4^ cells/well) before being incubated with NLRP3-IN-2, JC124, or MNS (0.5–20 μM) for 2 h and then assayed by a lactate dehydrogenase (LDH) assay kit (Beyotime Biotechnology, Shanghai, China).

### Inflammasome Activation

BMDMs were seeded in 24-well plates (1.0 × 10^6^ cells/well) and primed with LPS (100 ng/ml) for 4 h. After that, the cells were incubated with inflammasome inhibitors for 1 h, and then, the cells were treated with nigericin (5 μg/ml) for 30 min or ATP (5 mM) for 2 h or silica (100 μg/ml) for 6 h or MSU (100 μg/ml) for 6 h.

### Cytokine Analysis

The cell supernatant was collected after the activation of the inflammasome and centrifuged at 12,000 g for 1 min. Using relevant ELISA kits, IL-1β and TNF-α were assayed. Similar quality colon tissues were harvested from each group and incubated in DMEM. The culture supernatants were centrifuged for 1 min at 12,000 g and then IL-1β, IL-12p40, IL-6, and TNF-α levels were quantified by ELISA.

### Western Blotting

The cells were collected and lysed in lysis buffer. The sample lysates were sonicated and centrifuged at 12,000 g for 5 min, and the supernatant was collected. Protein samples were separated by sodium dodecyl sulfate-polyacrylamide gel electrophoresis and then transferred to nitrocellulose membranes. The membranes were blocked in 5% skim milk in TBS + Tween 20 (TBST) for 1 h at room temperature. After that, the membranes were incubated with primary antibodies (caspase-1, IL-1β, ASC, NLRP3, P65, P-P65, and β-actin) overnight at 4°C. The next day, after washing in TBST, the membranes were incubated with peroxidase-conjugated secondary antibody for 1 h at room temperature. Protein bands were visualized with a high-signal enhanced chemiluminescence Western blotting substrate (Tanon, China) using a Tanon-Chemi imaging system (Tanon-5200).

### Immunofluorescence Staining of ASC Specks

BMDMs (6 × 10^5^ cells/well) were placed in 24-well plates. After inflammasome activation, the cells were fixed in 4% paraformaldehyde for 20 min at room temperature, washed with PBS three times, followed by permeabilization with 0.2% Triton X-100, and blocked with 10% goat serum in PBS for 60 min. After that, we poured out the blocking solution and incubated with anti-ASC antibody (1:100) diluted in 5% goat serum in PBS at 4°C overnight. Then, BMDMs were washed with PBS, incubated with Alexa Flour 488 goat anti-rabbit IgG (Invitrogen, Carlsbad, United States) diluted in PBS (1:1000) for 2 h, and nuclei were stained with DAPI (Beyotime Biotechnology, Shanghai, China) for 1 h. The ASC-speck formation was imaged under a positive fluorescence microscope (Nikon, Japan).

### Animals and the DSS-Induced Colitis Model

Male C57BL/6 mice (weighing 25–27 g at 8–10 weeks old) were purchased from Charles River Laboratories (Beijing, China). They were kept in a 12 h reverse light/dark cycle, with *ad libitum* access to food and water.

The animals were divided into three groups. The control group (*n* = 5) received no DSS or inhibitors. The DSS group (*n* = 5) was treated with 3% DSS to induce colitis but no inhibitors. Animals in the DSS + MNS group were simultaneously treated with 3% DSS and MNS (30 mg/kg/day) for 5 days. According to a previously described protocol (Zaki et al., 2010), the disease activity index (DAI) was used to record weight loss, fecal diarrhea, and the presence of fecal blood. After 7 days, the mice were humanely killed by cervical dislocation. The colon tissue was excised for subsequent analysis. The animal experiments were conducted in strict accordance with the National Institutes of Health Guide for the Care and Use of Laboratory Animals, approved by the Ethics Committee of Ruijin Hospital, Shanghai Jiao Tong University School of Medicine.

### Histological Score

Approximately 1 cm of colon tissue was fixed in 4% formalin. The samples were then dehydrated and embedded in paraffin. Histological sections were stained with hematoxylin and eosin (H&E) and observed under an optical microscope (Nikon, Japan). Colitis severity was evaluated using a lesion score system. 1) Inflammation severity of the intestinal wall (0.5 = extremely mild; 1 = mild; 2 = moderate; 3 = intense inflammation). 2) Lesion severity (1 = mucosal layer; 2 = mucosa; 3 = submucosa or transmural injury). 3) Crypt damage (0 = no significant damage; 1 = 1/3 crypt damage; 2 = 2/3 crypt damage; 3 = crypt loss but surface epithelium intact; and 4 = crypt and surface epithelium missing). The scores were summed up to generate the final score. The average score [mean ± standard error of the mean (SEM)] of each animal in each group was calculated, and t-tests were performed among different groups.

### Myeloperoxidase Activity

Neutrophil infiltration into inflamed bowel mucosa was determined by an MPO activity assay using the O-dianisidine method (Qian et al., 2020). The protein extracted from colonic tissue was used to evaluate the MPO level according to the manufacturer’s instructions (Beyotime Biotechnology, Shanghai, China).

### Immunohistochemistry Staining

Colon tissue sections were deparaffinized, rehydrated, and blocked. Then, the sections were incubated with the following primary antibodies at 4°C overnight: Ly-6G (1:800), claudin-1 (1: 400), and MUC-2 (1:1000) overnight at 4°C. After that, the sections were incubated with an HRP Anti-Rabbit IgG antibody (1:800) for 1 h at room temperature. Positively stained sites were visualized by incubating with peroxidase-labeled streptavidin-complex (Beyotime Biotechnology, Shanghai, China), and the nuclei were counterstained with hematoxylin. The immune-positive sites were recognized by yellowish-brown staining, and the blue or purple staining showed the nuclei. The images were captured with a Nikon confocal microscope (Tokyo, Japan).

### Flow Cytometry

The colon tissue was collected when mice were killed and immediately minced and digested with collagenase IV (Sigma-Aldrich, MO, United States) 300 U/ml and a hyaluronidase solution (Sigma-Aldrich, MO, United States) 200 U/ml in Roswell Park Memorial Institute 1640 medium supplemented with penicillin and streptomycin and incubated at 37°C for 1 h. The cell suspension was centrifuged at 12,000 g for 5 min and mechanically dissociated through a sterile nylon mesh filter. For intracellular cytokine analyses, the cells were stimulated in complete medium plus a Cell Activation Cocktail and Brefeldin A for 4 h at 37°C. The assay antibodies included anti–FITC-CD45, APC-F4/80, Pe-Cy5-CD4, PE-IL-17A, APC-IFN-γ, PerCP-Cy5.5-CD11b, and also a Zombie NIR dye. Data were acquired on an LSR-II system (BD Biosciences) and analyzed using FlowJo software (V10, Becton Dickinson, Ashland, OR, United States).

### Statistical Analysis

Statistical analyses were conducted using two-tailed Student’s tests for comparisons between two groups and a one-way analysis of variance (ANOVA) followed by Dunnett’s test for comparisons between three or more groups. The results were expressed as the mean ± standard error of the mean (SEM). *p* < 0.05 indicated statistical significance. All analyses were carried out by GraphPad Prism v8.0 (La Jolla, San Diego, United States).

## Results

### The Effects of Inflammasome Inhibitors on IL-1β Secretion

**FIGURE 1:**
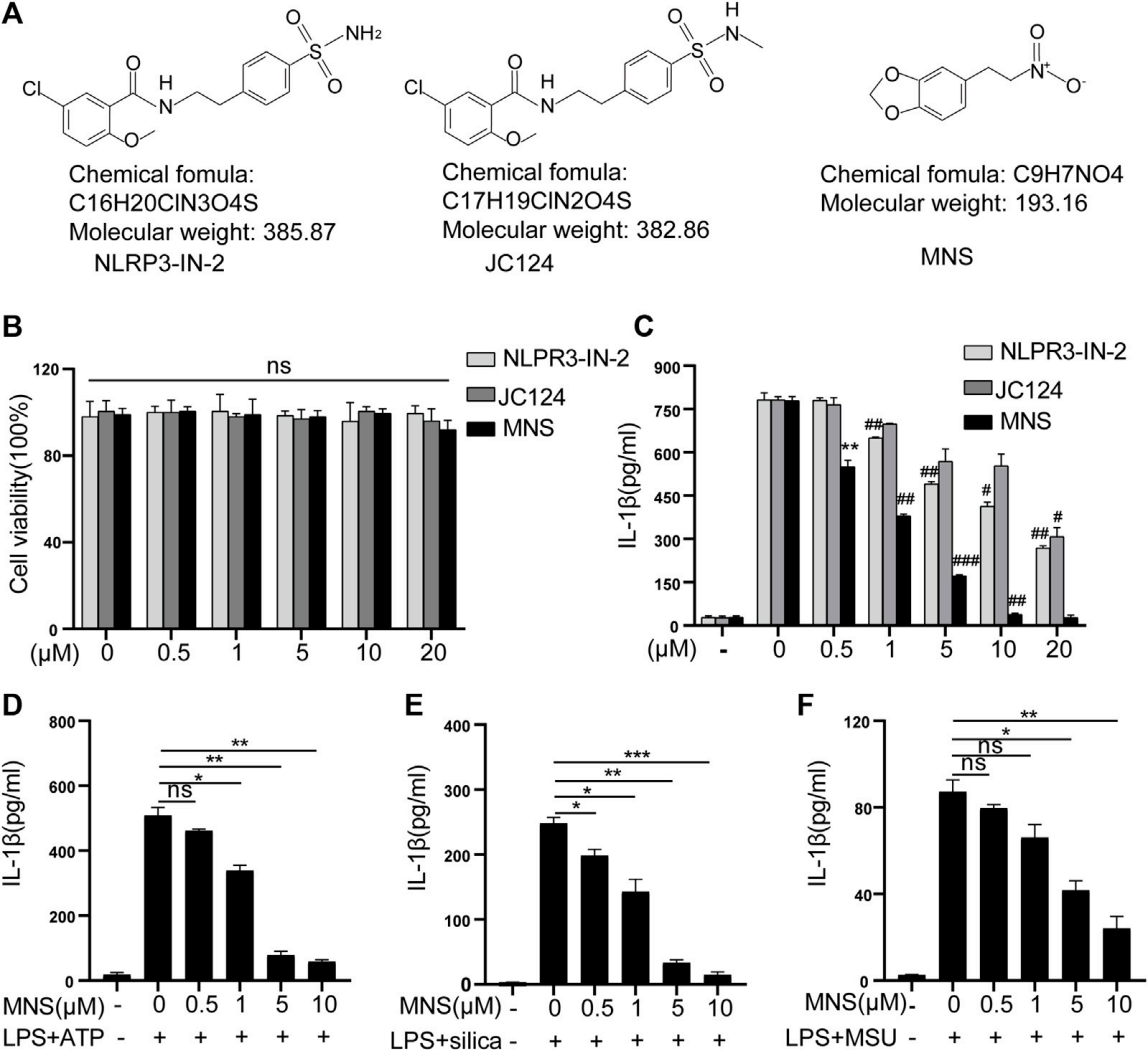
Inflammasome inhibitors inhibited IL-1β secretion. **(A)** Chemical structures of NLRP3-IN-2, JC124, and MNS. **(B)** BMDM viability was measured using the LDH assay after incubation with different doses of inflammasome inhibitors of 0, 0.5, 1, 5, 10, and 20 μM. **(C–F)** After LPS-primed BMDMs were pretreated with MNS (0.5–20 μM), IL-1β secretion was assayed by ELISA upon stimulation with nigericin (15 μM) for 30 min **(C)**, ATP (5 mM) for 2 h **(D)**, silica (100 μg/ml) for 6 h **(E)**, and MSU (100 μg/ml) for 6 h **(F)**. Data were expressed as the mean ± standard error of the mean (SEM) (*n* = 3). Statistical analyses were performed using an unpaired two-tailed Student’s t-test; ns = not significant, *p < 0.05, **p < 0.01, and ***p < 0.001 vs. without inhibitor treated; ^#^ *p* < 0.05, ^##^ *p* < 0.01, and ^###^ *p* < 0.001 vs. with inhibitor treated.

To assess the toxicity of NLRP3-IN-2, JC124, and MNS (Figure 1A) on BMDM viability, LDH assays were performed. Treatment with any of the three compounds up to 20 μM did not generate any cytotoxicity (Figure 1B). Thus, in the subsequent experiments, we used inhibitors at concentrations of 1–10 μM, a safe range for BMDM viability. NLRP3-IN-2, JC124, and MNS were added before NLRP3 inflammasome activation to investigate their possible inhibition. Of them, MNS showed significant inhibitory effects toward IL-1β production in a concentration-dependent manner (Figure 1C). To further confirm the inhibitory effects of MNS on the NLRP3 inflammasome, we stimulated BMDMs with ATP, silica, and MSU (monosodium urate crystals) separately. IL-1β secretion was significantly increased after using these stimulants, while it was decreased with the treatment of MNS in a dose-dependent manner (Figure 1D). These evidences suggested that MNS could inhibit IL-1β secretion effectively.

### MNS Inhibited Activation of the NLRP3 Inflammasome *In Vitro*

**FIGURE 2:**
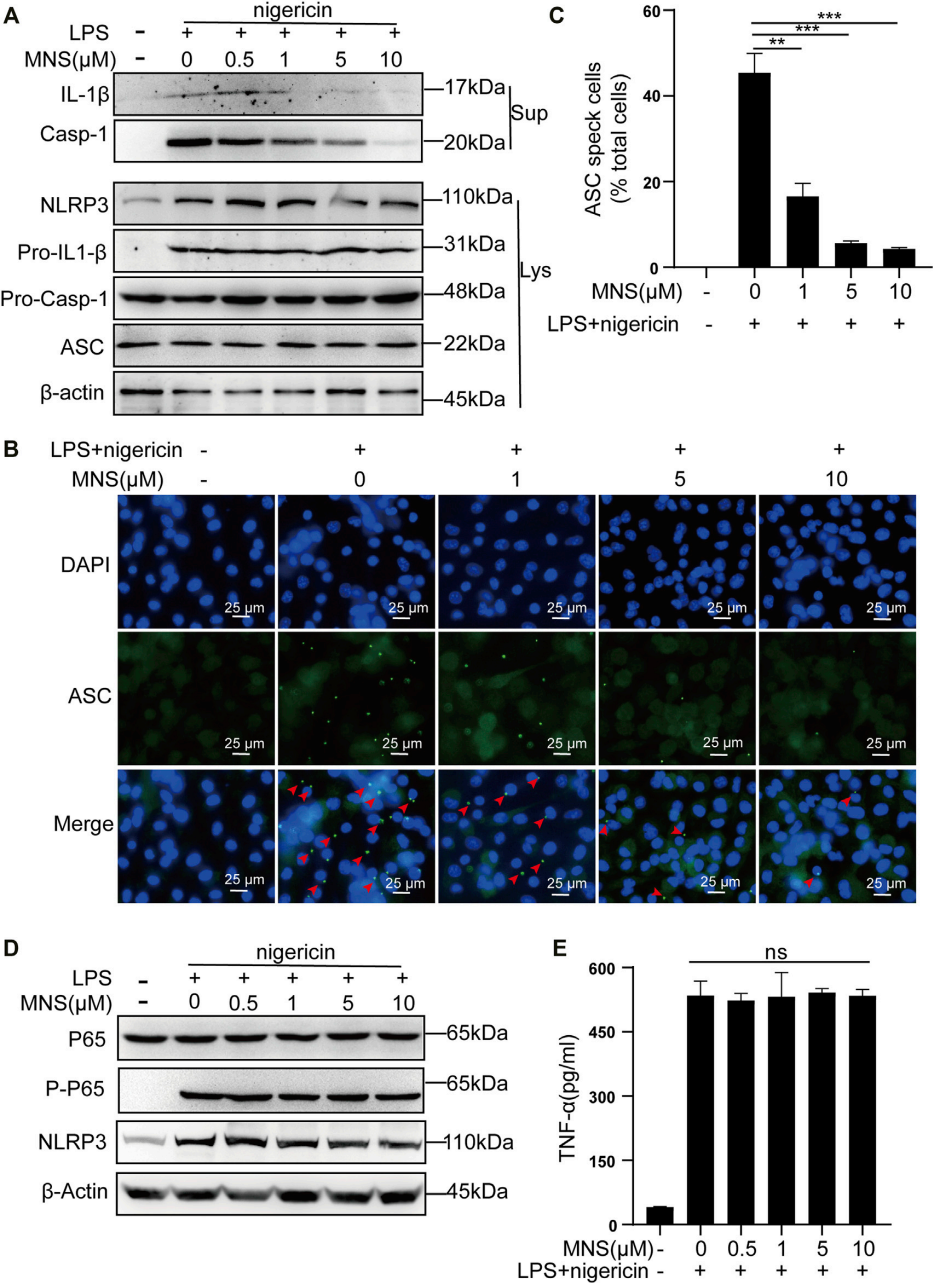
MNS inhibited activation of the NLRP3 inflammasome *in vitro.* **(A–E)** LPS-primed BMDMs were treated with MNS (0.5–10 μM) for 1 h, followed by 15 μM nigericin for 30 min. Mature IL-1β and caspase-1 (Casp-1) were measured in supernatants (Sup); NLRP3, pro–IL-1β, pro–caspase-1(Pro–Casp-1), and ASC were measured in whole-cell lysates (Lys) by immunoblotting **(A)**. BMDMs were stained with anti–ASC antibodies for ASC specks (green) and DAPI for nuclei (blue). ASC specks were marked with red arrows **(B)**. BMDMs with ASC specks were represented as a percentage of total cells **(C)**. BMDM p65 and P-p65 levels were examined by Western blotting. **(D)** TNF-α levels in the supernatant were determined by ELISA **(E)**. Data were expressed as the mean ± standard error of the mean (SEM) (*n* = 3). Statistical analyses were performed using an unpaired two-tailed Student’s t-test; ns = not significant; *p < 0.05, **p < 0.01, and ***p < 0.001 vs. without MNS treatment.

The inflammasome contains multiple proteins, and the expression of these proteins affects its activity in turn (Martinon et al., 2002). Therefore, we examined the protein levels of NLRP3, ASC, pro–IL-1β, pro–caspase-1 in cell lysates, and those of caspase-1 and its substrate IL-1β in cell supernatants by immunoblotting. Caspase-1 and IL-1β were reduced, while NLRP3, ASC, pro–IL-1β, and pro–caspase-1 remained unchanged (Figure 2A). ASC specks were observed upon ASC oligomerization, which thereby reflected NLRP3 inflammasome activation (He et al., 2014). To investigate whether MNS suppressed IL-1β release by reducing ASC aggregation, we performed immunofluorescence on ASC specks and observed that nigericin triggered ASC speck formation in macrophages in the nigericin group. Compared to this, ASC specks decreased significantly in the MNS-treated group in an MNS dose-dependent manner (Figures 2B,C; Supplementary Figure S1). Therefore, MNS could inhibit ASC speck formation. We next examined the important proteins of nuclear factor-κB (NF-κB), p65 and P-p65, which are the primary stages of NLRP3 inflammasome formation (Hayden and Ghosh, 2008). Western blotting showed that these protein levels were unchanged (Figure 2D). In addition, the effect of MNS on LPS-induced TNF-α secretion levels was investigated, and a negligible change in TNF-α levels was observed at MNS concentrations of 0.5, 1, 5, and 10 μM (Figure 2E). Collectively, the inhibitory effects of MNS on NLRP3 inflammasome activation were pronounced, whereas NF-κB expression was unaffected.

### MNS Relieved DSS-Induced Colitis Symptoms

**FIGURE 3:**
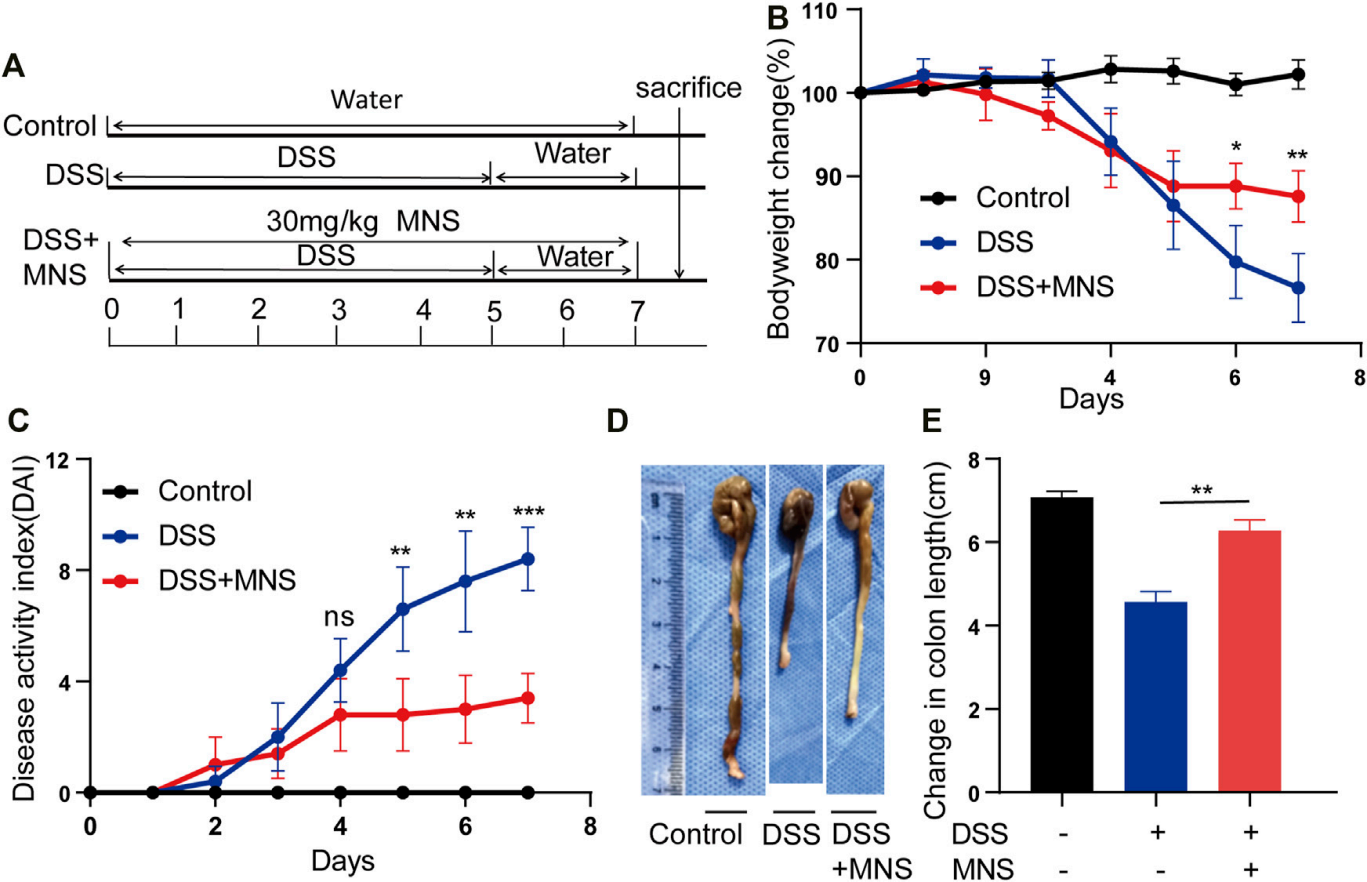
MNS relieved DSS-induced colitis symptoms. **(A)** Experimental DSS-induced colitis protocol. **(B)** Percentage of body weight loss in mice treated with DSS alone (blue line), DSS + MNS (red line), and Control mice (black line). **(C)** Disease activity index (DAI) in mice treated with DSS alone (blue line), DSS + MNS (red line), and Control mice (black line) over 7 days. **(D)** Gross colonic anatomy showing the shortening effects of MNS on DSS-induced colon. **(E)** Colon length was measured at autopsy. N = 3 mice/group. Data were presented as the mean ± standard error of the mean (SEM). In **(B,C,E)** *p < 0.05, **p < 0.01, and ***p < 0.001 vs. the DSS group.

To assess whether MNS protects against colitis *in vivo*, we designed an experiment using a DSS-induced colitis mouse model (Figure 3A). Both the DSS and DSS + MNS groups recorded weight loss on the fourth day, but with time, weight loss and disease activity index (DAI) in the DSS + MNS group improved compared with those in the DSS group (Figures 3B,C). We also observed a significant reduction in colonic length and splenomegaly in the DSS + MNS group compared to that in the DSS group (Figures 3D,E; Supplementary Figures S2A–C). In conclusion, our data suggest that MNS could significantly alleviate colitis symptoms of mice.

### MNS Reduced Histopathological Damage During DSS-Induced Colitis

**FIGURE 4:**
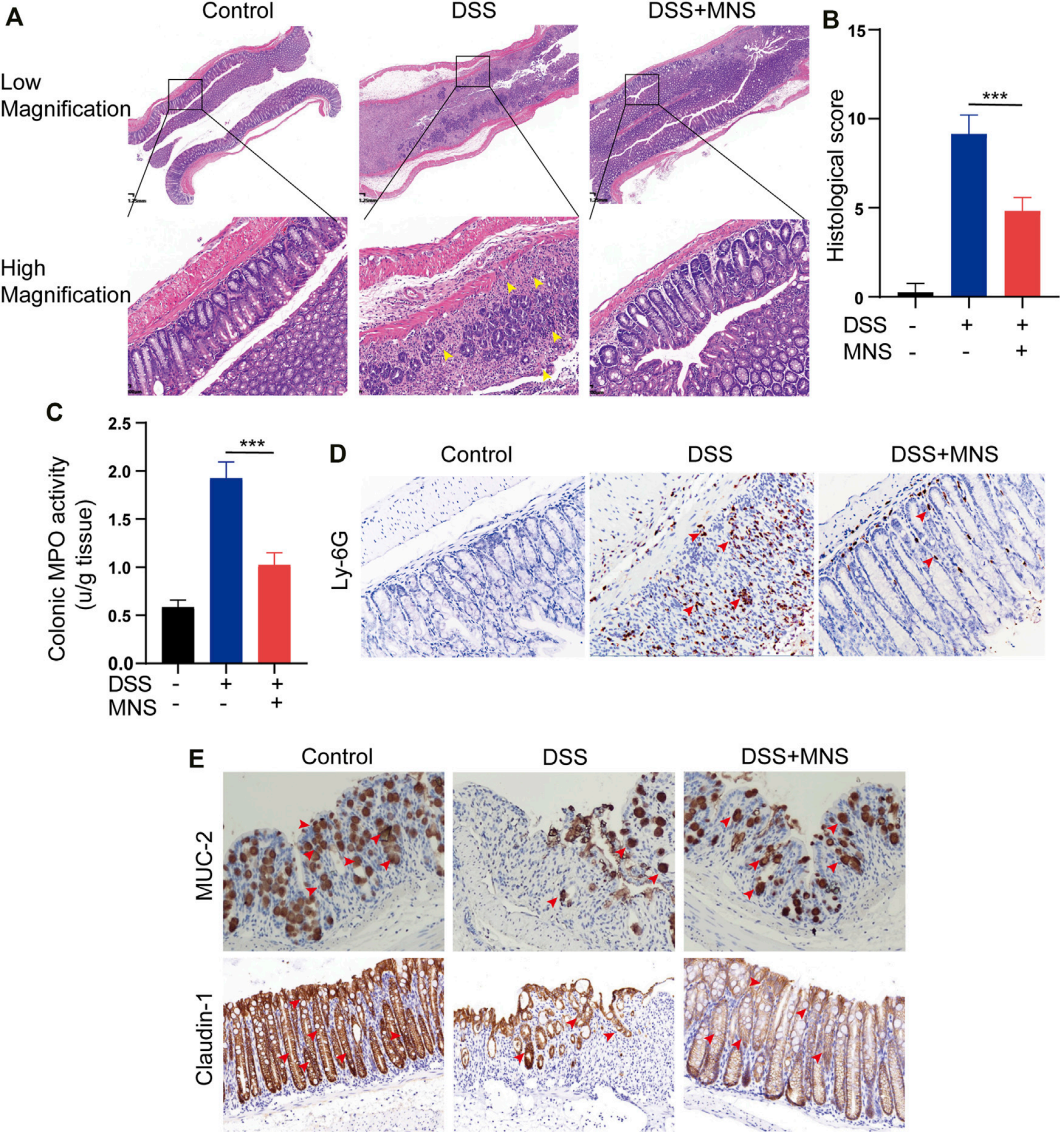
MNS reduced histopathological damage during DSS-induced colitis. **(A)** Hematoxylin and eosin stain of colon tissue, epithelium loss, and crypt structure collapse were marked with yellow arrows. **(B)** Histological scores of blinded sections. **(C)** Colon tissue culture supernatants were assayed for MPO. Immunohistochemistry staining of **(D)** Ly-6G **(E)** MUC-2, and claudin-1 in the colon were marked with red arrows. N = 3 mice/group. Data were presented as the mean ± standard error of the mean (SEM). ***p < 0.001 vs. the DSS group.

To observe the effects of MNS on intestinal tissue, we performed H&E staining on colon tissues from each group (Figure 4A). DSS treatment caused extensive colonic damage, accompanied by epithelium loss and crypt structure collapse compared with DSS + MNS treatment (Figures 4A,B). Myeloperoxidase (MPO) assay and immunohistochemical staining of Ly-6G were used to evaluate the recruitment and infiltration of neutrophils in mouse colon tissue. The results show that neutrophils significantly increased in the DSS group compared with the DSS + MNS group (Figures 4C,D). Colonic mucus is the first physical barrier that protects the colon against toxins and pathogenic invasion and is produced and maintained by glycosylated mucin-2 (MUC-2) and claudin-1 (Ambort et al., 2012; Citalán-Madrid et al., 2017; Chen et al., 2021). Immunohistochemistry staining revealed that the protein expression levels of MUC-2 and claudin-1 were significantly reduced in the DSS group, but the levels of these proteins in the DSS + MNS group were similar to those of the control (Figure 4E). Thus, MNS treatment retarded histopathological changes, protected the intestinal mucosa from damage, and maintained the intestinal barrier.

### MNS Attenuated Macrophage Infiltration and Regulated Cytokine Secretion of Mouse Colon With DSS-Induced Colitis

**FIGURE 5:**
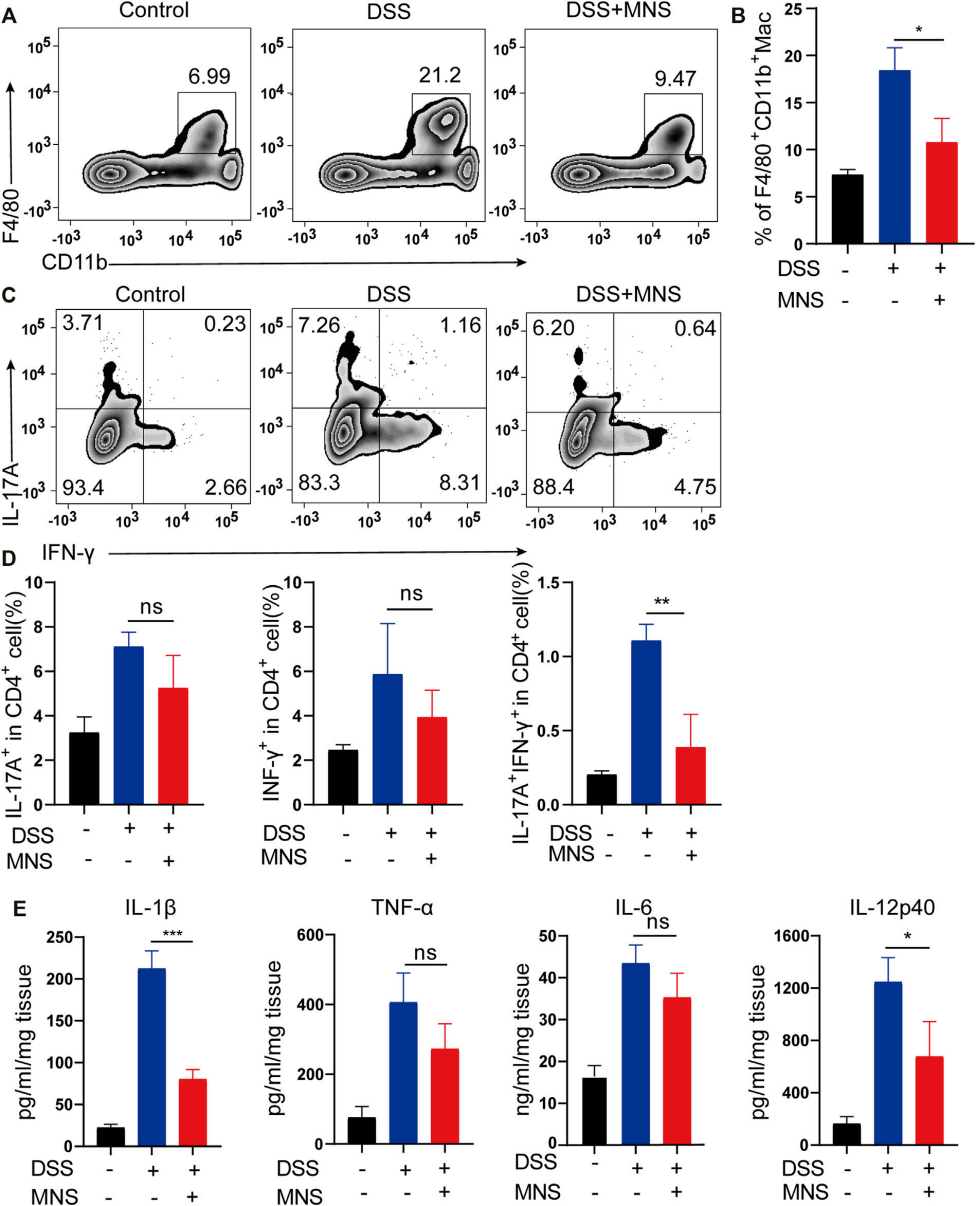
MNS attenuated macrophage infiltration and regulated cytokine secretion of mouse colon with DSS-induced colitis. **(A,B)** F4/80 + CD11b + labeled macrophages in infiltrated colon tissue were detected by flow cytometry. **(C)** IL-17A^+^IFN-γ^+^ helper T cells in colon tissue were analyzed by flow cytometry by IL-17A and IFN-γ intracellular staining. **(D)** Proportion analysis of IL-17A^+^, IFN-γ^+^, and IL-17A^+^IFN-γ^+^ cells in CD4^+^T cells. **(E)** Washed colon tissues were weighed and cultured *ex vivo* for 24 h, and the supernatants of colon biopsy were analyzed using TNF-α, IL-1β, IL-6, and IL-12p40 ELISA kit. N = 3 mice/group. Data were presented as the mean ± standard error of the mean (SEM); ns = not significant. *p < 0.05, **p < 0.01, and ***p < 0.001 vs. the DSS group.

In patients with IBD, considerable macrophages usually infiltrate the intestine (Bernardo et al., 2018; Wright et al., 2021). To explore this further, we used flow cytometry to examine the number and proportion of F4/80+ and CD11b+ labeled macrophages in colon tissues. Compared with the DSS group, the MNS + DSS group displayed reduced intestinal macrophage infiltration (Figures 5A,B). Secretion of macrophage-derived inflammatory cytokines, such as IL-1β, IL-12p40, and TNF-α, is required for T helper 17 (Th17) and T helper 1 (Th1) cell differentiation (Conforti-Andreoni et al., 2011). IL-17A is the marker of Th17, and IFN-γ is the marker of Th1 (Harbour et al., 2015), so we investigated the expression of IL-17A and IFN-γ by flow cytometry. The results showed that IL-17A^+^IFN-γ^-^CD4^+^ T cells (Th17) decreased slightly in the DSS + MNS group compared with those in the DSS group, but there was no significance (Figures 5C,D). However, IL-17A^-^IFN-γ^+^CD4^+^ T cells (Th1) and IL-17A^+^IFN-γ^+^CD4^+^ T cells decreased significantly in the DSS + MNS group when compared with the DSS group (Figures 5C,D). In addition, the levels of TNF-α, IL-1β, IL-6, and IL-12p40 in colon tissue were measured by ELISA, which were all reduced in DSS + MNS animals, and significance was only observed in IL-1β and IL-12p40 (Figure 5E). These data show that MNS attenuated macrophage infiltration and IL-17A^+^IFN-γ^+^CD4^+^ T cell differentiation and IL-1β, IL-12, and IL-6 secretion.

## Discussion

**FIGURE 6:**
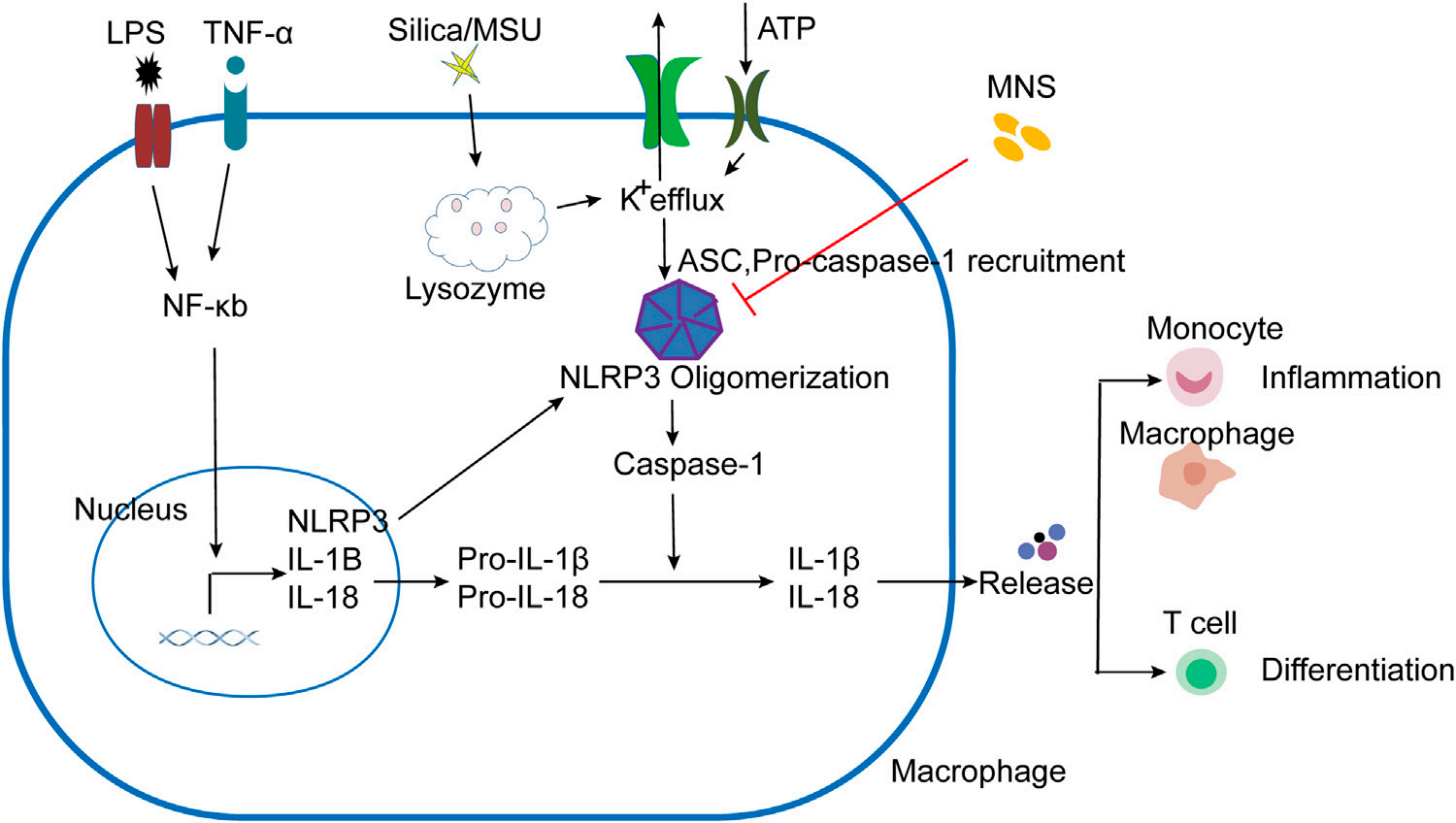
Schematic presentation of MNS-alleviated inflammatory responses. LPS/TNF-α promoted the activation of the NF-κB pathway, increased the transcription of NLRP3, Pro–IL-1β, and Pro–IL-18. NLRP3 responded to stimuli (ATP, MSU, and silica), induced K+ efflux, and ASC and pro–caspase-1 were recruited to assemble and form NLRP3 oligomerization with the active caspase-1. Pro-IL-1β and pro-IL-18 are cleaved into mature forms by caspase-1 following NLRP3 inflammasome activation. IL-1β and other cytokines were released to induce the aggregation of mononuclear/macrophages and Th cell differentiation. When administered with MNS, NLRP3 inflammasome activation was prevented and cytokine maturation release was reduced, thereby alleviating the inflammatory response.

IBD is a common clinical disease with a complex pathogenesis. Despite the continuous development of treatments, there are still limitations. Therefore, it is necessary to explore other effective and safe therapeutic drugs. As a cytoplasmic polyprotein complex and an important component of innate immunity, the NLRP3 Inflammasome has been studied by more and more researchers due to its vital role in immune response and diseases, including infectious diseases and metabolic diseases (Place and Kanneganti, 2019; Tartey and Kanneganti, 2020). It is known that the activation of the NLRP3 inflammasome is achieved through two sequential steps, termed as priming and assembly. The activation of Toll-like receptor 4 (TLR4) by lipopolysaccharide (LPS) provides the priming signal (signal 1), thereby increasing the transcription of NLRP3, pro–IL-1β, and IL-18 through the NF-κB–mediated pathway (Huang et al., 2017; Mangan et al., 2018). Further stimulation by infections and/or tissue damage (e.g., ATP, MSU, and silica) (signal 2) leads to a series of responses, such as K^+^ efflux (Mariathasan et al., 2006; Martinon et al., 2006; Dostert et al., 2008), that trigger the assembly of NLRP3 inflammasomes following NLRP3 inflammasome activation (Liu et al., 2014) (Figure 6). We have confirmed that MNS has a broad-spectrum inhibitory effect on ATP, MSU, and silica-induced inflammasome activation *in vitro*. As the core of the inflammatory response, the NLRP3 inflammasome may provide new targets for the treatment of various inflammatory diseases. This study is the first description of using compound-MNS on enteritis mice with DSS-induced intestinal inflammation and explored its inhibitory effects on the NLRP3 inflammasome, which may function as an effective therapeutic for IBD.

He et al. identified MNS as one of the most potent inhibitors for NLRP3 inflammasome activation by screening a kinase inhibitor library in 2014 (He et al., 2014). Since then, several studies have confirmed its anti-inflammatory effects on NLRP3, and no effect on NLRC4 or AIM2 inflammasome (He et al., 2014; Xiao et al., 2016; Patel et al., 2018). In addition to reducing the release of mature IL-1β, MNS could also induce other cellular responses, such as cell death and IL-18 maturation, suggesting that directly targeting NLRP3 inflammasome activation may be more effective than blocking IL-1β alone. Several previously identified inhibitors of the NLRP3 inflammasome, such as Bay11-7082 and Parthenolide, acted upstream of the inflammasome pathway and inhibited the activation of the NF-κB pathway, thereby affecting NLRP3 ATPase activity, ASC oligomerization, and reducing IL-1β and TNF-α production (Saadane et al., 2007; Juliana et al., 2010), while recent studies reported that anti-TNF-α agents, such as infliximab, adalimumab, and ustekinumab, could result in severe side effects (Cohen and Sachar, 2017). Therefore, inhibiting upstream molecules of the inflammasome pathway may not be an ideal choice. Our experiments showed that MNS did not affect the activation of NF-κB or the release of TNF-α, suggesting that MNS may have fewer side effects in IBD treatment.

It was also reported that MNS could inhibit Syk kinase that could modulate the activity of the ASC-containing inflammasome in macrophages by regulating the formation of ASC specks (Hara et al., 2013), which was consistent with our results. Interestingly, MNS’s inhibitory effects on Syk kinase could inhibit platelet aggregation, which was also consistent with some reports of many antiplatelet drugs having good anti-inflammatory effects (Nannizzi-Alaimo et al., 2003; Friedman et al., 2009; Patil et al., 2010; Petrovic et al., 2020). For example, as the current traditional IBD therapeutic agent, sulfasalazine has been proven to have an anti-platelet aggregation effect (Patel et al., 2012). Considering that MNS may play a role through multiple pathways in the treatment of IBD, which makes it a better therapeutic option.

As synthetic small-molecule inhibitors of the NLRP3 inflammasome, NLRP3-IN-2, JC124, and MNS can all reduce IL-1β secretion in BMDMs. However, in our study, NLRP3-IN-2 and JC124 were not as effective as reported by Marchetti et al. (2014); Kuwar et al. (2019), and MNS was the most effective. The reason for this may be due to different drug sensitivities to different cell lines. Here, we used primary BMDMs because they were superior to tumor cell lines and more closely resembled autologous macrophages, which would make the results more meaningful.

Inflammatory cytokines play an important role in the development of IBD. Clinical studies have shown that the concentration of IL-1β dramatically increased in the serum of IBD patients and inflamed colonic tissues appeared (McAlindon et al., 1998; Ludwiczek et al., 2004). Along with this, mature IL-1β production was also significantly upregulated in our mouse model of DSS-induced colitis. Studies have shown that excessive IL-1β can lead to increased intestinal permeability, promote dendritic cell and macrophage activation, and facilitate the accumulation of IL-17A secreting cells (Al-Sadi et al., 2012; Harbour et al., 2015). Our study showed that MNS administration significantly relieved mouse colitis and decreased IL-1β secretion and macrophage infiltration. Meanwhile, other inflammatory factors, such as IL-12, were also decreased upon MNS treatment. Previous studies have shown that IL-1β and IL-12 can promote the differentiation of helper T cells (Mahida, 2000; Wang et al., 2021), and T helper 17 (Th17) cells are required for the development of IBD (Geremia et al., 2014; Harbour et al., 2015; Alexander et al., 2022). IFN-γ–deficient Th17 cells maintained their Th17 phenotype but failed to induce colitis in mouse models, and IL-17A^+^IFN-γ^+^CD4^+^ T cells were considered to be more pathogenic (Harbour et al., 2015; Tian et al., 2021). During DSS-induced colitis, IL-17A^+^IFN-γ^+^CD4^+^ T cell levels were significantly reduced after MNS treatment. In the development of colitis, macrophages secrete inflammatory cytokines such as IL-1β and IL-12, leading to inflammatory cell aggregation, increasing inflammatory cytokine secretion, and promoting Th cell differentiation, thereby exacerbating immune response [30] (Figure 6). From this point, inhibiting inflammatory cytokine secretion or IL-17A^+^IFN-γ^+^CD4^+^ T cell differentiation may slow down or limit immune-mediated intestinal damage, which may provide a theoretical basis for IBD immunotherapy in the future.

There are still some limitations in our study. First, MNS is the NLRP3 inflammasome inhibitor, which has the same drug target as the classic inflammasome inhibitor MCC950. The effect of MNS can be further compared with that of MCC950; second, how MNS affects intestinal microflora and microenvironment changes needs to be further verified.

In conclusion, this is the first report showing that MNS reduced intestinal tissue damage, attenuated macrophage infiltration, and regulated IL-1β and IL-12 pro-inflammatory cytokine secretion in the mouse colon with DSS-induced colitis. It also impacted the contribution of IL-17A^+^IFN-γ^+^CD4^+^ T cell differentiation. It is more efficient in inhibiting the secretion of interleukin-1β (IL-1β) than NLRP3-IN-2 and JC124. Therefore, MNS may serve as a potential treatment for inflammatory bowel disease in humans.

## Data Availability

The original contributions presented in the study are included in the article/Supplementary Material; further inquiries can be directed to the corresponding authors.
