# Supplementary material for: 3,4-Methylenedioxy-β-Nitrostyrene Alleviates Dextran Sulfate Sodium–Induced Mouse Colitis by Inhibiting the NLRP3 Inflammasome
Source: Front Pharmacol. 2022 Jun 15;13:866228. doi: 10.3389/fphar.2022.866228 (PMC9240698; doi:10.3389/fphar.2022.866228)
Supplement: Supplementary file 1 [file DataSheet1.PDF]

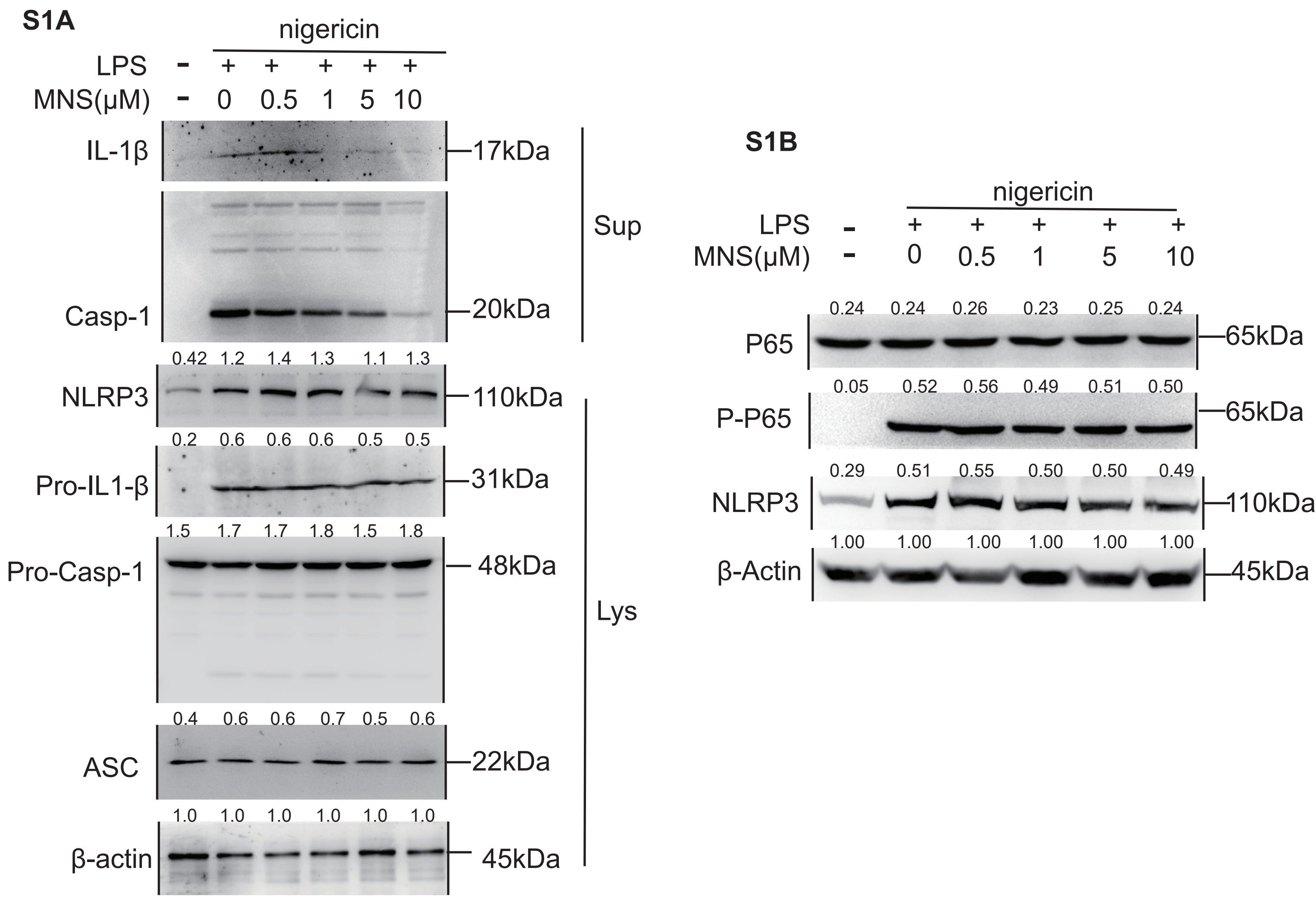

Figure S1.Bone marrow-derived macrophages (BMDMs) were primed with LPS (100ng/ml) for 4 hr. The cells were treated with MNS for 1 hour and then stimulated with nigericin (15μg/ml) for 30 min, the cell culture supernatants and cell lysates were immunoblotted for (A) pro-caspase-1, caspase-1, pro-IL-1β, and IL-1β, ASC, NLRP3; (B) P65, P-P65, NLRP3..

S2A

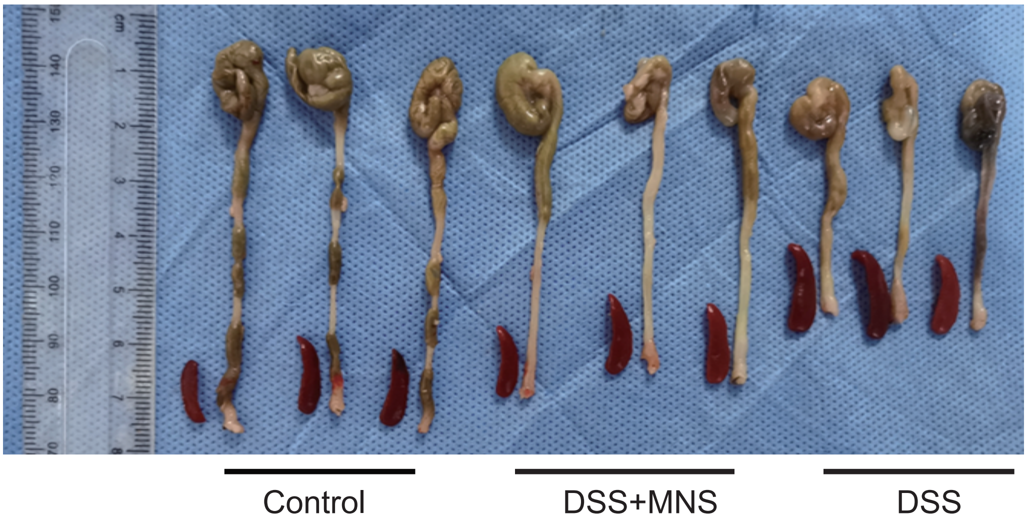

S2B

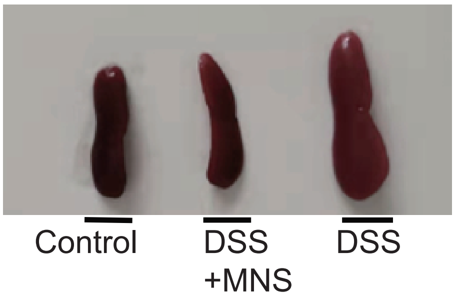

S1C

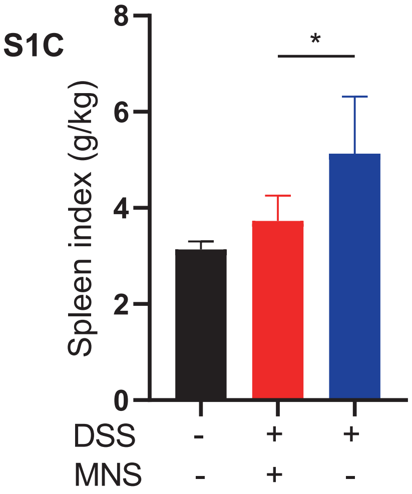

Figure S2. (A) Colon length of mice was measured at autopsy. (B) Macroscopic appearances of spleens. (C) Spleen weight index. One representative experiment of three is shown. 3 mice per group. Data were expressed as the mean ± standard error of the mean (SEM) (n = 3). *p < 0.05 vs. the DSS group.
